# Supplementary figures and images for: Galactofuranose (Galf)-containing sugar chain contributes to the hyphal growth, conidiation and virulence of F. oxysporum f.sp. cucumerinum
Source: PLoS One. 2021 Jul 30;16(7):e0250064. doi: 10.1371/journal.pone.0250064 (PMC8323920; doi:10.1371/journal.pone.0250064)

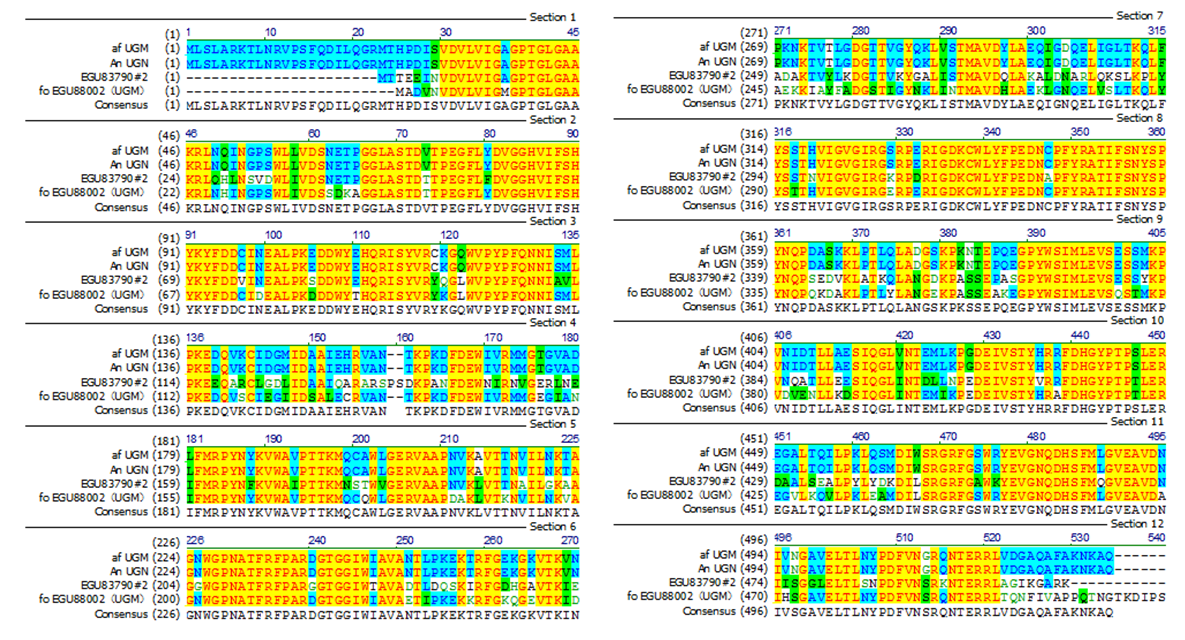

Supplement: S1 Fig — BLASTp searches of the A. fumigatus UDP-galactopyranose mutase GlfA/Ugm1 (AFU_3G12690) in the genome of F. oxysporum 5176 identified three homologous genes, ugmA (EGU88002.1) and ugmB (EGU83790). The ugmA encodes a predicted protein (519 amino acids) sharing 80% of identity with A. fumigatus GlfA, while the ugmB encodes a protein (507 amino acids) sharing 71% of identity with A. fumigatus GlfA. (TIF) [file pone.0250064.s001.tif]

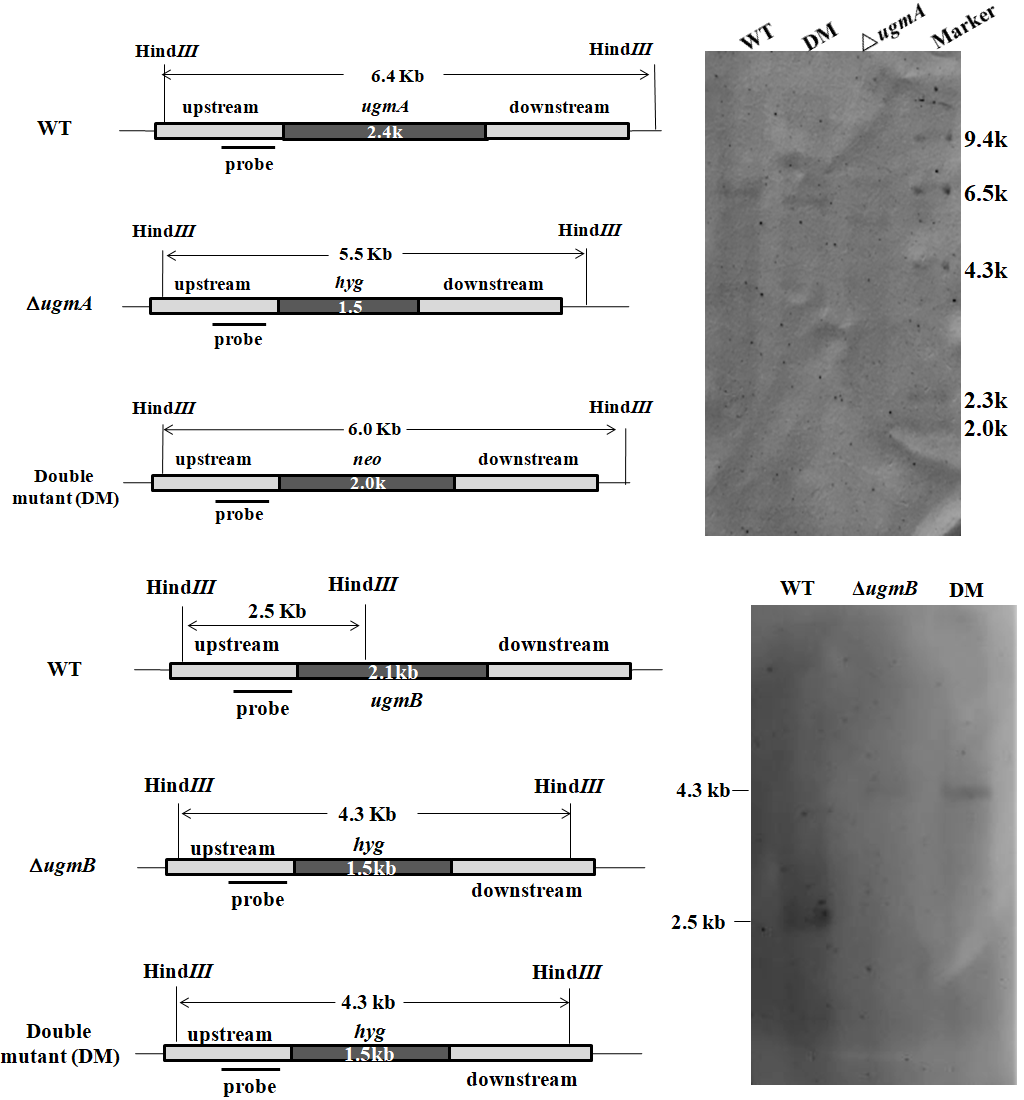

Supplement: S2 Fig — Southern blotting was carried by detecting HindIII-digested fragments with a probe in the upstream non-coding region of the ugmA or ugmB gene. (TIF) [file pone.0250064.s002.tif]

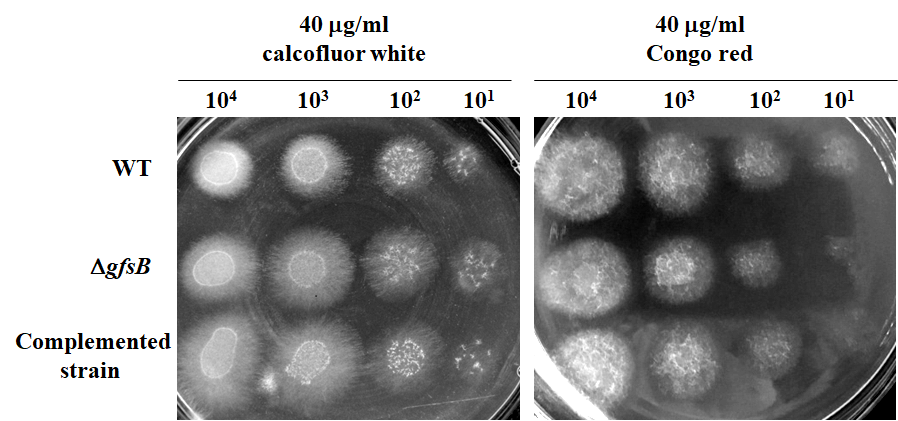

Supplement: S3 Fig — A serial dilution of conidia of 1×10^4^−1×101 of the WT, the mutant and complemented strain were inoculated on PDA agar plates supplemented with 40 μg/ml calcofluor white or 40 μg/ml Congo red. Strains were cultured at 28°C. (TIF) [file pone.0250064.s003.tif]
