# Supplementary material for: Galactofuranose (Galf)-containing sugar chain contributes to the hyphal growth, conidiation and virulence of F. oxysporum f.sp. cucumerinum
Source: PLoS One. 2021 Jul 30;16(7):e0250064. doi: 10.1371/journal.pone.0250064 (PMC8323920; doi:10.1371/journal.pone.0250064)
Supplement: S1 Table — (PDF) [file pone.0250064.s004.pdf]

S1 Table. Oligonucleotides used in this study.

**Primer**	Sequence 5'-3'	Experimental Use
ugmA u1	AATAGTTTCAAGGTCGGTTTACATGAGC	Upstream flanking of *ugmA*
ugmAh2	GCTCCTTCAATATCATCTTCTGTCGCATAAAAATGTCAGTTTTGCAGCG	Upstream flanking of *ugmA*
HygA1	CGCTGCAAAACTGACATTTTTATGCGACAGAAGATGATATTGAAGGAGC	Hygromycin
HygA2	CGAGGCGCTAACTCGCTCTCCACAAAGTGATTTCAGTAACGTTAAGTG	Hygromycin
ugmA d 1	CACTTAACGTTACTGAAATCACTTTGTGGAGAGCGAGTTAGCGCCTCG	Downstream flanking of *ugmA*
ugmA d 2	AGTCAACCATATTCTCCATCGAGTAGTC	Downstream flanking of *ugmA*
ugmB u1	CTGAAAGGGAGTATTACTATTCGGTC	Upstream flanking of *ugmB*
ugmB u2	CTCCTTCAATATCATCTTCTGTCCCTGGGACAGGTTAAATCAGC	Upstream flanking of *ugmB*
hyroB 1	GCTGATTTAACCTGTCCCAGGGACAGAAGATGATATTGAAGGAG	Hygromycin
hygroB 2	CAACTCGGACATAGATAATGGATTTCAGTAACGTTAAGTGGATCC	Hygromycin
ugmB d2	CTTCGTGCTGCTCGGTCTGGTTGATGTTC	Downstream flanking of *ugmB*
ugmA U1	AATAGTTTCAAGGTCGGTTTACATGAGC	Upstream flanking of *ugmA*
ugmA n2	GAATGGAAATTGTAAGCGTTAATCTAGAGCATAAAAATGTCAGTTTTGCAGCGG	For double mutant
neo U1	CCGCTGCAAAACTGACATTTTTATGCTCTAGATTAACGCTTACAATTTCCATTC	neomycin
neo U2	CGTTTCTCGAGGCGCTAACTCGCTCTCCACAAAGTTCTAGAGAATAGGAACTT	neomycin
ugmA n d1	AAGTTCCTATTCTCTAGAACTTTGTGGAGAGCGAGTTAGCGCCTCGAGAAACG	for double mutant
ugmA d2	AGTCAACCATATTCTCCATCGAGTAGTC	downstream flanking
ugmA F	AGTAACCGCTGCAAAACTGAC	Confirmation of Δ*ugmA* mutant
ugmA R	GCTAACTCGCTCTCCACAAAG	Confirmation of Δ*ugmA* mutant
ugmA U-1	TCATCTCGTTGCTCCAGCATG	Confirmation of Δ*ugmA* mutant
Hygro	TACTGCTTACAAGTGGGCTGATC	Confirmation of Δ*ugmA* mutant
ugmA C-F	CCCaagcttAGGTCCGGTGTAAGCACATC	complementation of Δ*ugmA*
ugmA C-R	AAAActgcagTTTCTTGTACACATGTCTCTTG	complementation of Δ*ugmA*
UgmB u1	GAGAGTATGTTGAACCAAACTTGGGCCGATAC	Confirmation of ΔugmB mutant
Hygro	TACTGCTTACAAGTGGGCTGATC	Confirmation of Δ*ugmB* mutant
ugmA U-1	TCATCTCGTTGCTCCAGCATG	Confirmation of double mutant
Neo G-1	AGTTGGTGACGGATCGAGTG	Confirmation of double mutant
Dr-ugmA u 3'p	GAGCCTGTGTGTAGAGATACAAGGTTTCTTGTACACATGTCTCTTGAG	*ugmA* revertant in

		double mutant
A Phleo 5’p	CTCAAGAGACATGTGTACAAGAAACCTTGTATCTCTACACACAGGCTC	*ugmA* revertant in double mutant
A Phleo 3’p	GCGCTAACTCGCTCTCCACAAAGTTCTAGAAAGAAGGATTACCTCTAAAC	*ugmA* revertant in double mutant
Dr-ugmA d 5’p	GTTTAGAGGTAATCCTTCTTTCTAGAACTTTGTGGAGAGCGAGTTAGCGC	*ugmA* revertant in double mutant
DrugmB u 3’p	GAGCCTGTGTGTAGAGATACAAGGTGACCGGTGACAACTCGGACATAG	*ugmB* revertant in double mutant
B Phleo 5’p	CTATGTCCGAGTTGTCACCGGTCACCTTGTATCTCTACACACAGGCTC	*ugmA* revertant in double mutant
B Phleo 3’p	GACAACTCGGACATAGATAATGTCTAGAAAGAAGGATTACCTCTAAACAAG	*ugmA* revertant in double mutant
Dr-ugmB d 5’p	CTTGTTTAGAGGTAATCCTTCTTTCTAGACATTATCTATGTCCGAGTTGTC	*ugmA* revertant in double mutant
s-ugmA5'p	ATAGGCATAATTCTTGAACGG	Probe for Δ*ugmA*
s-ugmA3'p	AGAACTTCGGAAAATGATGG	Probe for Δ*ugmA*
s-ugmB 5,p	CATGAAAGGGAGTATTACTATT	Probe for Δ*ugmB*
s-ugmB 3’P	AAGTCTCATTTCGTGCTCAGTCTG	Probe for Δ*ugmB*
EF1α-1	TCCTTCCAACGTCACCACTG	Real-time PCR
EF1α-2	TGAAAGAAGCGGCACCCATAG	Real-time PCR
ACT1F	TTGAAAGATGGCAGATGGTG	Real-time PCR
ACT1R	CTATGTGAACTTATGTATTGAGCAGC	Real-time PCR
ugmA 5’	AGTTACCAGCGACCTTGTTG	Real-time PCR
ugmA 3’	TCATGCGACCTTACAACTAC	Real-time PCR
ugmB 5’	ATAACTTCAAGGTCTGGGC	Real-time PCR
ugmB 3’	AGAATAGCGTTGGTAGTCAC	Real-time PCR
gfsAu5'p	ATTGTACTCGTCTGATTCCAATAGCCATTC	Upstream flanking of *gfsA*
gfsAu3'p	GCTCCTTCAATATCATCTTCTGTCCCCGCGAGTAAATTGACCCCTGTTC	Upstream flanking of *gfsA*
gfsAH5’p	GAACAGGGGTCAATTTACTCGCGGGGACAGAAGATGATATTGAAGGAGC	Hygromycin
gfsAH3’p	CGGGAAAAGGAAAGGCTCATTAGCGATTTCAGTAACGTTAAGTGGATC	Hygromycin
gfsAd5’p	GATCCACTTAACGTTACTGAAATCGCTAATGAGCCTTTCCTTTTCCCG	downstream flanking of *gfsA*
gfsAd3'p	GAGCTCTATGCGAAGGAAAGTCTGTGTCATG	downstream flanking of *gfsA*
gfsA C-F	GGGGTACC ACTCTGTTAGCAGTTGGACTCTCC	complementation of Δ*gfsA*
gfsA C-R	CGGAATTCATTATTATCTACAAAACCAGTCTATC	complementation of Δ*gfsA*
gfsA u-1	ACCACAATGGATGCCATCATCG	Confirmation of Δ*gfsA* mutant
Hygro	TACTGCTTACAAGTGGGCTGATC	Confirmation of Δ*gfsA* mutant
s-gfs 5'p	ATCCTCAGTCGATTTGTTCTTG	Probe for Δ*gfsA*
s-gfs 3'p	ATAATTGGCTCATCGCTGG	Probe for Δ*gfsA*
